# Artificial intelligence assisted detection of large vessel occlusion on CT angiography in acute stroke patients: a multi-reader multi-case study

**DOI:** 10.1093/bjrai/ubaf001

**Published:** 2025-02-07

**Authors:** Kiruba Nagaratnam, Phil Mathieson, Anna Podlasek, Peter Slade, Gary A Ford, Anthony Cox, James H Briggs, Zoe V J Woodhead, George Harston

**Affiliations:** Royal Berkshire NHS Foundation Trust, Reading, RG1 5AN, United Kingdom; Oxford University Hospitals NHS Trust, Stroke Medicine, Oxford, OX3 9DU, United Kingdom; Division of Radiological and Imaging Sciences, University of Nottingham, Nottingham, NG7 2UH, United Kingdom; Taysie Innovation MedTech Ecosystem (TIME), University of Dundee, Dundee, DD2 1FD, United Kingdom; Stroke Medicine Morriston, Morriston Hospital, Swansea, SA6 6NL, United Kingdom; Oxford University Hospitals NHS Trust, Stroke Medicine, Oxford, OX3 9DU, United Kingdom; Radcliffe Department of Medicine, University of Oxford, Oxford, OX3 9DU, United Kingdom; North Bristol NHS Trust, BS10 5NB, United Kingdom; Royal Berkshire NHS Foundation Trust, Reading, RG1 5AN, United Kingdom; Brainomix Limited, Oxford, OX2 7HN, United Kingdom; Brainomix Limited, Oxford, OX2 7HN, United Kingdom; Oxford University Hospitals NHS Trust, Stroke Medicine, Oxford, OX3 9DU, United Kingdom; Brainomix Limited, Oxford, OX2 7HN, United Kingdom

**Keywords:** artificial intelligence, AI, acute ischemic stroke, CT angiography, CTA, large vessel occlusion, LVO, collateral score

## Abstract

**Objectives:**

We assessed the impact of artificial intelligence (AI) software (e-CTA, Brainomix) on clinical decision-making in patients with suspected acute ischemic stroke.

**Methods:**

A retrospective, multi-reader-multi-case crossover design compared readers’ performance with vs without software support. Twenty cases were included, 10 with large vessel occlusion (LVO) and 10 without LVO. Twenty-one NHS clinicians, representing intended software users ranging in experience, conducted 2 sessions (washout period > 2 weeks). In session 1, software support was provided for 10 randomly selected cases. In session 2, support allocation was reversed. Outcome measures included LVO detection, collateral scoring, diagnosis, treatment decision, time taken and confidence.

**Results:**

Sensitivity, specificity, and accuracy of LVO detection improved with imaging software for LVO detection, with increased confidence and reduced time taken. There was no significant difference in collateral scoring or diagnoses.

**Conclusion:**

e-CTA can improve performance of NHS clinicians when interpreting acute stroke imaging.

**Advances in knowledge:**

This paper provides new evidence that AI decision support software has the capacity to improve the performance of representative users in the NHS when interpreting imaging to identify patients for acute stroke treatments.

## Introduction

In current clinical practice, cervical and intracranial CT angiography (CTA) is the primary method for detecting large vessel occlusion (LVO) in patients with suspected stroke. CTA determines mechanical thrombectomy (MT) eligibility by identifying LVO in the anterior and posterior circulation.^1–3^ Collateral blood flow is an important predictor of outcome from LVO stroke and can be used to approximate the salvageable brain tissue and gauge the suitability for transport between hospitals; hence, collateral status derived from CTA is an important component of the final decision on whether to proceed with MT for the particular patient.^1^^,^^3–5^ The speed of treatment delivery is critically important to maximize the chance of good patient outcomes^6–8^; therefore, there is a need for real-time, fast and reliable imaging interpretation to identify and localize the occlusion and guide treatment decisions. However, access to neuroimaging expertize is not readily available in all hospitals and even in specialist centres may not be available out of hours.^9^ Artificial intelligence (AI) decision support tools are increasingly used to support clinicians in the timely identification of patients eligible for MT.^10–12^ AI decision support software is recommended to be used in routine clinical practice by both UK (Stroke—Getting It Right First Time—GIRFT) and US guidelines.^13^

Regulatory and clinical evidence for AI imaging software is typically based on standalone performance testing against expert consensus. A growing number of papers have examined the standalone performance of software devices for automated detection of proximal anterior circulation LVOs^14–20^ (typically defined as intracranial internal carotid artery [ICA] or middle cerebral artery [MCA] M1 occlusions), including some with very large datasets^21^ or with challenging, real-world datasets.^22^^,^^23^ Performance of automated tools for assessment of collaterals has also been explored.^24–26^ In practice, however, these software devices are designed to generate a triage notification or to be used as a decision-support tool. While standalone performance is important, so too is the impact that the software outputs have on clinical decision making. There is much more limited literature evaluating the impact of such software impact on clinicians’ imaging interpretation,^27^ or on clinical outcomes.^28–31^

This multiple reader multiple case (MRMC) study aims to measure the impact of decision support software for CTA (“Brainomix 360 e-CTA,” Brainomix Limited) on clinical decision-making in the context of patients presenting to acute hospitals with a clinically suspected stroke in the UK. A range of outcome measures were assessed (reflecting the different features of the software), relating to LVO detection, collateral scoring, diagnosis, treatment decisions, time taken, and confidence. The modest sample size (20 cases and 21 readers) was justified pragmatically, positioning the study as a relatively small discovery study with exploratory hypothesis testing. Stroke clinicians with a range of experience from different UK institutions were included to provide a representative sample of the software users across the NHS. The study’s overall objective was to assess whether AI imaging improves accuracy, speed and confidence in CTA image interpretation in NHS stroke clinicians.

## Methods

### Procedure

This clinical performance assessment utilized a MRMC approach to evaluate the performance of a set of clinical readers when interpreting CTA imaging for a selection of patients with LVO of the MCA or intracranial ICA (*N* = 10) or without LVO (*N* = 10). All cases were randomly selected from a historical research registry of cases with suspected stroke undergoing CTA.^25^

**Figure 1.**
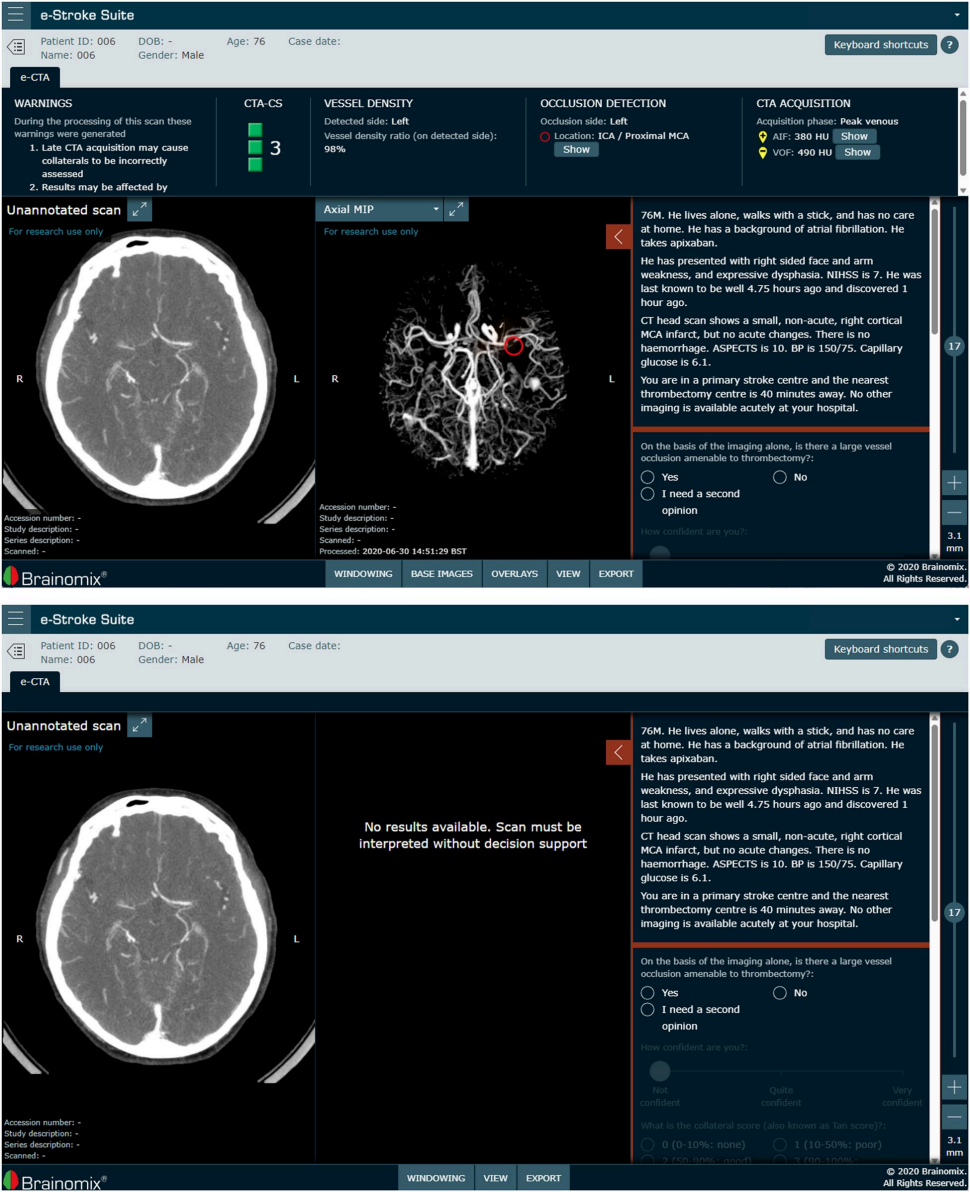
Screenshot examples of the study viewing platform with (top) and without (bottom) decision support from e-CTA.

A viewing platform was created to view CTA images alongside representative clinical vignettes for each case and to collect readers’ responses. When images were presented without decision support (“without e-CTA”), only the unannotated CTA images (and vignette) were presented. When images were presented with decision support from e-CTA (“with e-CTA”), the annotated output from the automated e-CTA analysis was also presented in the form of an axial maximum intensity project and details of the estimated CTA collateral score (CS), vessel density and occlusion detection. Figure 1 below shows screenshot examples of the viewing platform *with* (top) and *without* (bottom) decision support from e-CTA.

**Figure 2.**
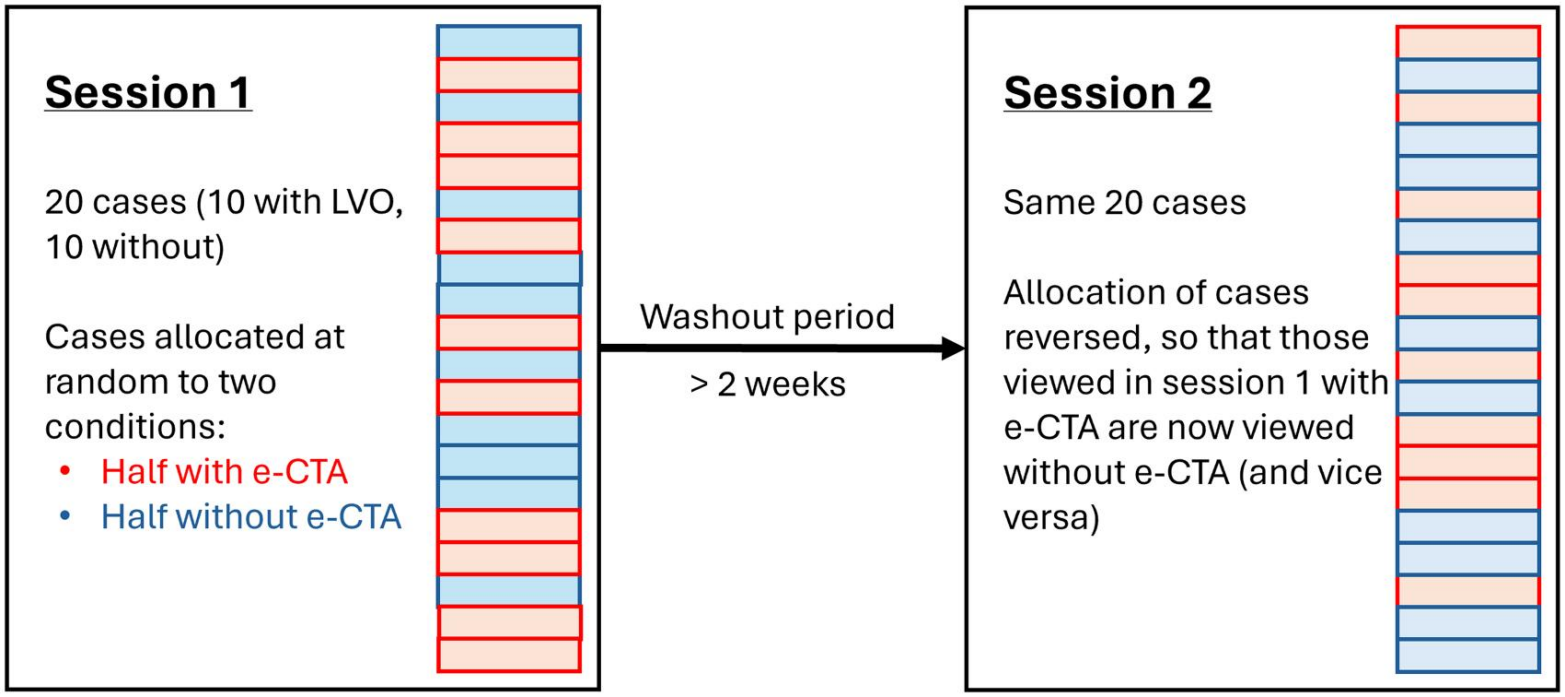
Schematic depiction of the study procedure, showing the 2 sessions, spaced by at least 2 weeks, each containing 20 cases.

Readers viewed the 20 images in 2 sessions, spaced by at least 2 weeks. Figure 2 shows a schematic depiction of the study procedure. At the first session, half of the cases were randomly selected to be presented with e-CTA and the remaining half were presented without e-CTA. The selection of cases randomized separately for each reader to avoid bias. At the second session, the allocation of decision support was reversed so that by the end of the second session, all cases had been reviewed once with and once without decision support from e-CTA. The readers’ ability and confidence to detect key diagnostic features (LVO and CS), and to arrive at a diagnosis and treatment decision was recorded. A radiological ground truth was set by an expert neuroradiologist with access to follow-up information. Questions that readers were asked are outlined in Table 1.

**Table 1.** Questions and response options for readers.

Question	Response options
On the basis of the imaging alone, is there a large vessel occlusion amenable to thrombectomy?	Yes
	No
	I need a second opinion
How confident are you regarding LVO?	Sliding scale from 0 (Not confident) to 100 (Very confident)
What is the collateral score (Tan score)?	0 (0%-10%; none)
	1 (10%-50%; poor)
	2 (50%-90%: good)
	3 (90%-100%: complete)
How confident are you about the collateral score?	Sliding scale from 0 to 100
On the basis of the imaging and the vignette, what is the most likely diagnosis?	Stroke
	Seizure
	Migraine
	Other
How confident are you about the diagnosis?	Sliding scale from 0 to 100
What treatment would you offer (in the absence of other contraindications)? (select one or two answers)	IV Thrombolysis
	IV Thrombolysis; only after additional imaging
	Mechanical thrombectomy
	Mechanical thrombectomy; only after additional imaging
	None

### Case selection

Twenty cases were randomly selected from a historical research registry of cases with suspected stroke undergoing CTA.^25^ A balanced mix of cases with and without LVO (10 of each) were selected.

All cases met the following criteria: (1) age 18 years or over; (2) availability of baseline CTA with adequate quality (eg, free from excessive motion artefact); (3) availability of demographic information and follow-up imaging; and (4) no evidence of intracranial haemorrhage.

Selection of LVO cases was enriched to ensure that occlusion locations matched the distribution in the HERMES meta-analysis that informed the guidelines for patient selection for MT.^32^ LVO was defined as an occlusion of either the intracranial ICA or proximal segment of the MCA. Patients with distal occlusions or posterior circulation stroke were not included, as these are beyond the scope of the e-CTA software.

The CT images were acquired from a range of scanners with different protocols, representative of the range of data acquired in clinical practice. An experienced stroke physician (G.H.), independent of the test reader group, generated representative clinical vignettes. All image headers and clinical vignettes were anonymized.

### Readers

Twelve readers completed all 20 cases at both sessions; a further 9 completed only the first session. Data from all 21 were included in the final analysis.

The readers were representative UK clinicians, specialising in either stroke, geriatric medicine, or radiology, at a specialist trainee (ST) or consultant grade (see Table 2).

**Table 2.** Clinical role and specialty of the study readers (where available), and number of cases read with and without e-CTA decision support.

Reader	Cases With e-CTA	Without e-CTA	Role	Specialty
Reader01	20	20	ST	Radiology
Reader02	20	20	Consultant	Stroke
Reader03	20	20	Consultant	Stroke
Reader04	20	20	Consultant	Stroke
Reader05	20	20	Consultant	Geriatric medicine
Reader06	20	20	Consultant	Stroke
Reader07	20	20	Consultant	Geriatric medicine
Reader08	20	20	Consultant	Stroke
Reader09	20	20	Consultant	Stroke
Reader10	20	20	Consultant	Geriatric medicine
Reader11	20	20	Consultant	Stroke
Reader12	20	20	Consultant	Stroke
Reader13	8	12	Consultant	Stroke
Reader14	7	13	Consultant	Stroke
Reader15	11	9	ST	Stroke
Reader16	10	10	Consultant	Radiology
Reader17	8	12	ST	Stroke
Reader18	9	11	Consultant	Stroke
Reader19	11	9	ST	Stroke
Reader20	8	12	Consultant	Stroke
Reader21	10	10	Consultant	Stroke

ST = specialist trainee.

Readers were familiarized with the purpose of the study and received training in the use of e-CTA and the viewing portal prior to beginning the study. The training consisted of written materials and a webinar video describing the purpose and function of the software, as well as the CS grading system. The design of the study was described, including instruction that occlusions (if present) would be restricted to the anterior circulation.

### Ground truth

Ground truth for presence or absence of LVO and diagnosis was set by an expert neuroradiologist with access to follow up clinical information, final diagnosis, and follow up imaging. Due to the subjective nature of collateral scoring, a consensus ground truth was taken for CSs between 3 expert clinicians: 2 neuroradiologists, and 1 stroke physician.

### Automated CTA analysis

Images on the reader platform were processed with Brainomix 360 e-CTA, CE-marked decision support software. The software provides analysis and viewing capabilities of CTA datasets in stroke patients, including assessment of collateral status, detection of intracranial ICA or MCA LVOs, and visualization of intracranial blood vessels. Image analysis involves the following: (1) image preprocessing, including brain extraction and normalization (registration); (2) blood vessel segmentation and generation of a maximum intensity projection (MIP) image or multiplanar reconstructions (MPRs); (3) detection of occlusions in the patent MCA large vessels through use of a convolutional neural network (CNN); (4) generation of a probabilistic heatmap of reduced vessel density through comparison of affected and unaffected hemispheres; and (5) quantification of a CTA CS^33^ (CTA-CS) on the basis of the vessel density map.

On the reader platform, the outputs of e-CTA were presented, including the preprocessed CTA image and the MIP, overlaid with the vessel density heatmap, the CTA-CS and the location of any LVO detected by the software (see Figure 1). Information on the acquisition phase (as detected by the software) is also shown to facilitate user interpretation of image quality.

### Statistical analysis

A number of outcome measures were collected for each reader and analysed as described in Table 3. Each analysis compared the impact of e-CTA support on the outcome measure.

**Table 3.** Outcome measures and analysis methods.

Measure	Description	Analysis
LVO AUC	Area under the ROC curve for LVO detection	OR-DBM analysis
LVO sensitivity	Sensitivity of LVO detection (TP/(TP+FN)	OR-DBM analysis
LVO specificity	Specificity of LVO detection (TN/(TN+FP)	OR-DBM analysis
LVO accuracy	Percentage accuracy of LVO detection	Mann Whitney U
LVO confidence	Mean confidence in LVO detection	Mann Whitney U
CS accuracy	Percentage accuracy of collateral scores	Mann Whitney U
CS confidence	Mean confidence in collateral scoring	Mann Whitney U
Diagnosis accuracy	Percentage accuracy in diagnosis	Mann Whitney U
Diagnosis confidence	Mean confidence in diagnosis	Mann Whitney U
Treatment confidence	Mean confidence in treatment decision	Mann Whitney U
Time taken	Time taken to complete all scores per case	Mann Whitney U

LVO = large vessel occlusion; CS = collateral score; TP = true positive; FN = false negative; TN = true negative; FP = false positive; OR-DBM = Obuchowski-Rockette/Dormann Berbaum and Metz.

The outcome measures for LVO detection (sensitivity, specificity and area under the receiver operating characteristic curve [AUC]) were analysed using the unified Obuchowski and Rockette/Dorman, Berbaum and Metz (OR-DBM) MRMC methods devised by Hillis et al,^34^ as implemented in the MCMCaov package in R.^35^ This analysis computed the overall area AUC, sensitivity and specificity for LVO detection at the group level.

For the remaining measures, percentages (for accuracy) or averages (for confidence and time taken) were calculated for each reader in the with and without e-CTA conditions. The results for each condition were then compared using Mann-Whitney U tests (a non-parametric equivalent of an independent samples t-test). A non-parametric test was chosen as the outcome measures were either bounded variables (eg, percentages), or because data exploration showed that they were not normally distributed. An independent samples test was chosen as not all cases were reviewed twice (once with and once without) by each reader.

In addition to the outcome measures listed above, the agreement between readers and ground truth (with and without e-CTA) was assessed using Cohen’s Kappa.

An alpha criterion of *P* < .05 was used to indicate statistical significance.

#### LVO detection sensitivity analysis

For LVO detection, readers were given the options “Yes,” “No” or “I need a second opinion.” If the reader selected the latter option, the response was coded as an incorrect response; that is, either a false negative (for positive cases) or a false positive (for negative cases). In order to evaluate the impact of this coding scheme, a sensitivity analysis was performed whereby the LVO analyses (for LVO AUC, sensitivity, specificity, accuracy, confidence, and Cohen’s kappa) were repeated, but excluding any cases where a response of “I need a second opinion” was given.

#### Treatment decision

The frequencies of readers’ decisions to recommend treatment with IV thrombolysis and/or MT (either with or without further imaging) were evaluated.

## Results

**Figure 3.**
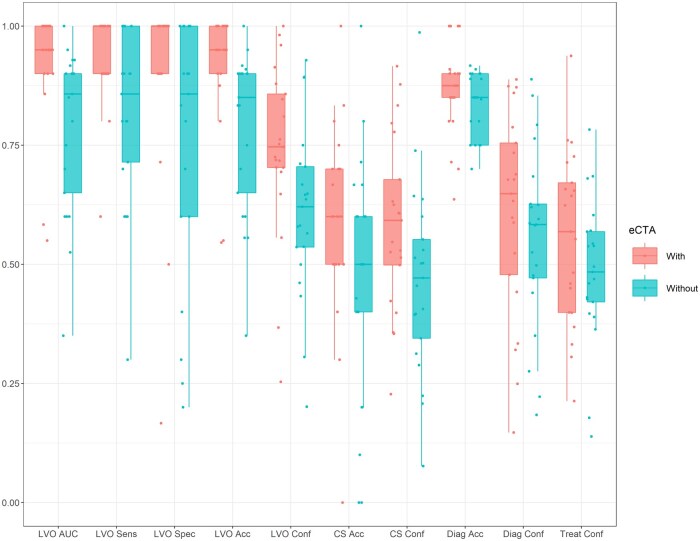
Boxplot showing group level summary statistics (median and interquartile range) for each outcome measure (LVO AUC, LVO sensitivity, LVO specificity, LVO accuracy, LVO confidence, CS accuracy, Collateral Score confidence, Diagnosis accuracy, Diagnosis confidence, and Treatment confidence; for a description of each outcome measure, see Table 3), when readers viewed cases with (red) or Without (blue) e-CTA decision support. Outcome measures for each reader are shown in red and blue points.

**Figure 4.**
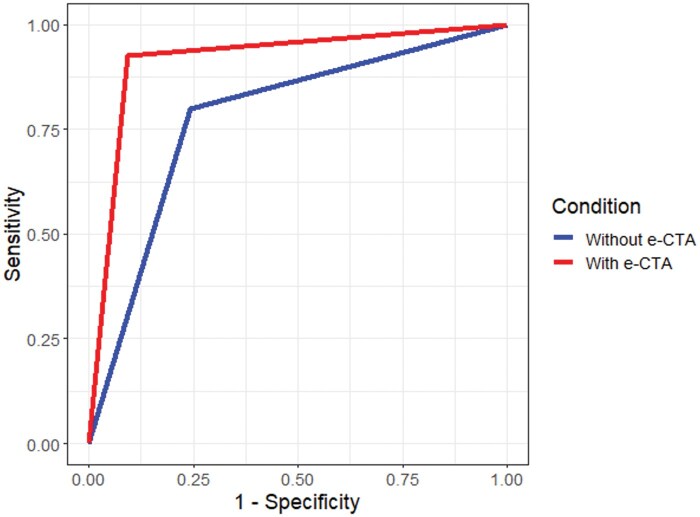
ROC curves for LVO detection of readers in the with e-CTA (red) and without e-CTA (blue) conditions.

Table 4 and Figure 3 show the total number of cases reviewed with or without e-CTA, the mean of each outcome measure with and without e-CTA. A ROC curve for the LVO detection measure is shown in Figure 4.

**Table 4.** Summary statistics for each outcome measure are reported for cases reviewed with and without e-CTA decision support.

Measure	With e-CTA	Without e-CTA	*p*
N Cases	322	338	NA
LVO TP/FP/TN/FN	149/15/146/12	135/41/128/34	NA
LVO AUC	0.92 [CI: 0.86, 0.98]	0.79 [CI: 0.69, 0.88]	*t* = 4.35, *p* < .001
LVO sensitivity	0.94 [CI: 0.88, 1.00]	0.82 [CI: 0.68, 0.96]	*t* = 2.95, *p* < .001
LVO specificity	0.91 [CI: 0.81, 1.00]	0.75 [CI: 0.63, 0.88]	*t* = 2.75, *p* = .012
LVO accuracy	0.95 [IQR: 0.90, 1.00]	0.85 [IQR: 0.65, 0.90]	*U* = 350, *p* < .001
LVO confidence	0.75 [IQR: 0.70, 0.86]	0.62 [IQR: 0.54, 0.70]	*U* = 327, *p* = .007
CS accuracy (%)	0.60 [IQR: 0.50, 0.70]	0.50 [IQR: 0.40, 0.60]	*U* = 282, *p* = .124
CS confidence	0.59 [IQR: 0.50, 0.68]	0.47 [IQR: 0.34, 0.55]	*U* = 303, *p* = .038
Diagnosis accuracy (%)	0.88 [IQR: 0.85, 0.90]	0.85 [IQR: 0.75, 0.90]	*U* = 262, *p* = .303
Diagnosis confidence	0.65 [IQR: 0.48, 0.75]	0.58 [IQR: 0.47, 0.63]	*U* = 266, *p* = .261
Treatment confidence	0.57 [IQR: 0.40, 0.67]	0.48 [IQR: 0.42, 0.57]	*U* = 35, *p* < .001
Time taken (s)	125 [IQR: 106, 146]	135 [IQR: 113, 164]	*U* = 441, *p* < .001

For LVO AUC, sensitivity and specificity, the results are the estimated group means, shown with 95% CI in brackets. For all other outcome measures, medians are reported with IQR in brackets. Results with and without e-CTA are compared using either t-tests or Mann Whitney U tests as appropriate. LVO = large vessel occlusion; CS = collateral score; TP = true positive; FN = false negative; TN = true negative; FP = false positive.

The results demonstrate that all outcome measures were numerically higher (ie, better performance) when the readers were given e-CTA decision support, than when they were not. Using the pre-specified alpha criterion of *p* < .05, a statistically significant difference was observed for LVO AUC, LVO sensitivity, LVO specificity, LVO accuracy, LVO confidence, CS confidence, treatment confidence and time taken. Confidence levels for LVO detection, collateral scoring and treatment decisions all improved by around 10% when e-CTA support was available. The time taken to review the cases also showed a small but significant improvement of ∼10 s per case. The results for CS accuracy, diagnosis accuracy and diagnosis confidence were not statistically significant.

For LVO detection *with* e-CTA, Cohen’s Kappa was 0.83 (95% confidence interval [CI]: 0.77, 0.89), indicating very good agreement between readers and ground truth. For LVO detection *without* e-CTA, Cohen’s Kappa was 0.56 (95% CI: 0.47, 0.64), indicating moderate agreement. Overall accuracy improved by around 10% with use of e-CTA although this varied between readers (range: −10% to 44%).

### LVO detection sensitivity analysis

A sensitivity analysis was conducted to evaluate the effect of the way LVO detection responses were coded when readers selected “I need a second opinion.” This response was selected 18 times when e-CTA support was available (6% of responses), and 42 times when e-CTA support was not available (12% of responses). In the main analysis, “I need a second opinion” responses were treated as incorrect responses.

A second analysis was conducted where the “I need a second opinion” cases were excluded from the analysis. This changed the results as shown in Table 5. As shown in the table, excluding these cases had the effect of reducing the number of false positive (FP) and false negative (FN) counts.

**Table 5.** Alternative results for LVO detection sensitivity and specificity, where cases with the response of “I need a second opinion” were omitted from the analysis.

Measure	With e-CTA	Without e-CTA	*p*
*N* cases included	304	296	NA
*N* cases omitted	18	42	NA
LVO TP/FP/TN/FN	149/6/146/3	135/13/128/20	NA
LVO AUC	0.96 [CI: 0.93, 1.00]	0.88 [CI: 0.79, 0.97]	*t* = 2.21, *p* = .03
LVO sensitivity	0.98 [CI: 0.96, 1.00]	0.88 [CI: 0.76, 0.99]	*t* = 2.22, *p* = .03
LVO specificity	0.94 [CI: 0.87, 1.00]	0.88 [CI: 0.75, 1.00]	*t* = 1.01, *p* = .32
LVO accuracy	1.00 [IQR: 0.95, 1.00]	0.90 [IQR: 0.85, 0.95]	*U* = 346, *p* < .001
LVO confidence	0.76 [IQR: 0.70, 0.88]	0.65 [IQR: 0.54, 0.71]	*U* = 328, *p* = .006

TP = true positive; FN = false negative; TN = true negative; FP = false positive.

The results of the second analysis were broadly similar to the main analysis. As in the main analysis, the difference between with and without e-CTA conditions were significant for LVO AUC, sensitivity, accuracy and confidence; however, the difference was not significant for LVO specificity in this analysis. For the second analysis, Cohen’s Kappa was 0.94 (95% CI: 0.9, 0.98) with e-CTA, indicating very good agreement. Without e-CTA, Cohen’s Kappa was 0.78 (95% CI: 0.71, 0.85), indicating good agreement.

### Treatment decision

The readers’ decisions to recommend treatment with IV thrombolysis and/or MT (either with or without further imaging, in the context of the imaging and clinical information provided in the vignette) was also examined (Table 6). This showed that treatment recommendations were higher when e-CTA was available than when it was not.

**Table 6.** Rates of treatment recommendations (in percentage) for ground truth data, and for cases viewed with e-CTA vs without e-CTA.

Condition	IVT	IVT after imaging	MT	MT after imaging
With e-CTA	38%	21%	26%	33%
Without e-CTA	36%	17%	20%	33%

IVT = intravenous thrombolysis; IVT after imaging = intravenous thrombolysis only after further imaging; MT = mechanical thrombectomy; MT after imaging = mechanical thrombectomy only after further imaging.

## Discussion

This study demonstrates a significant improvement in performance of readers in the detection of LVO on CTA with AI decision support software compared to without. As well as showing an improvement in AUC, sensitivity, specificity, and accuracy, the decision support software also resulted in a higher degree of confidence in reader findings.

This increase in reader confidence is also reflected in the reduction in the proportion of cases where readers required a second opinion to determine whether LVO was present, occurring twice as often when e-CTA support was not available. Moreover, readers were more likely to recommend treatment, both in the form of IV thrombolysis and MT, when they reviewed the scans with decision support than without.

Use of e-CTA also resulted in improvements in readers’ confidence in collateral scoring. An improvement in collateral scoring accuracy was observed, but this was non-significant likely due to the modest sample size (10 cases with LVO) and distribution of CSs. Readers were significantly faster to assess cases when e-CTA support was available—the time taken measure encompassed the total time to assess the presence or absence of LVOs, the CS, the diagnosis and treatment decision. Diagnosis accuracy did not change significantly, but performance on this measure was high in both with and without conditions (88% and 85%, respectively).

This study included a range of readers from different clinical specialty backgrounds, and of varying levels of experience, reflecting the different models delivering acute services for stroke patients at different sites, and at different times, across the NHS. These results suggest that in addition to improving individual readers’ performance, decision support software reduces the range of performance across the sample user group. This may have particular benefit in reducing the variability of care between different hospitals, and at different times.

As well as assessing the performance of users in a research setting, this study also investigated the relationship between decision support software and reader confidence and the time taken for clinical assessment and decision making. These are important outcomes for any healthcare technology when considering any real-world impact, as clinician confidence is an important element in determining the speed at which treatment can be delivered. This is especially important in care of acute stroke patients, in whom a reduced time to treatment has been shown to improve outcomes. Treatment with MT is highly efficacious, and research has shown that treating more acute stroke patients with MT will result in better long term outcomes (ie, fewer patients with serious disability) and lower long term healthcare costs to the economy.^36^

It is important to note that in order for AI software to be integrated into routine clinical practice, users need to be trained in the correct use of the software, and to be made aware of the opportunities and limitations of its use. Reader studies such as this one can help build the understanding of both intended use and impact. Ultimately, the responsibility for clinical decisions lies with the clinician using the software. Nevertheless, it is important that the software has been appropriately evaluated and any risks identified before deployment that might influence patient care, hence the need for a robust regulatory framework to guide the use of AI decision support software.

This study is consistent with and complementary to the growing body of research demonstrating the benefits of AI-driven decision support software for interpretation of acute stroke imaging, both in terms of MRMC reader (with-or-without) studies,^27^^,^^37–39^ and studies evaluating the impact of AI software on clinical outcomes.^28–31^ Future studies will evaluate the impact of e-CTA in real-world prospective patient cohorts. Once such example has now been published, assessing the LVO detection performance of e-CTA in a prospective study.^40^

Limitations of this study include the relatively small number of scans assessed, and incomplete assessment by some readers. The study was not designed or powered to allow evaluation of the standalone performance of the software (ie, the accuracy of the outputs of the software relative to ground truth), but details of this can be found in 2 recent studies.^16^^,^^17^ The proportion of cases with LVO is higher than typically seen in an unselected population of patients presenting with acute stroke symptoms where 20%-25% patients have LVO. The LVO cases were limited to MCA or ICA occlusions only, and did not include cases with distal or posterior circulation occlusions. Together with the synthetic vignettes and the low numbers, the population does not reflect real-world prevalence of the conditions, but was designed to balance statistical power against the pragmatic consideration of time required for clinicians to participate in the study.

## Conclusions

AI decision support software has the capacity to improve the performance of representative users in the NHS when interpreting imaging to identify patients for acute stroke treatments. When using the software, clinicians were more likely to make recommend treatment with either systemic thrombolysis or MT. Prospective studies are ongoing to capture the impact of e-CTA technology in a real-world setting, without the bias inherent in a retrospective, enriched dataset. Future algorithm development could focus on broadening the applicability of the software to distal or posterior circulation strokes.

The results of this real-world study demonstrate the potential for AI based decision support software to improve accuracy and confidence of UK clinicians in detection of LVO, as well as reducing variability of performance between individuals and increasing clinician confidence. These outcomes could help deliver more consistent, expeditious, and accurate selection of patient for MT, with the eventual aim of improving long term clinical outcomes for patients.
